# High level of D‐dimer predicts ischemic stroke in patients with infective endocarditis

**DOI:** 10.1002/jcla.23206

**Published:** 2020-02-03

**Authors:** Nan Xu, Yakun Fu, Shuanglin Wang, Shenghui Li, Dong Cai

**Affiliations:** ^1^ Department of Infectious Diseases Tianjin Medical University General Hospital Tianjin China; ^2^ NHC Key Laboratory of Hormones and Development (Tianjin Medical University) Tianjin Key Laboratory of Metabolic Diseases Tianjin Medical University Chu Hsien‐I Memorial Hospital & Tianjin Institute of Endocrinology Tianjin China; ^3^ Department of Cardio‐Thoracic Surgery Tianjin Medical University General Hospital Tianjin China; ^4^ Department of Neurosurgery Key Laboratory of Post‐Neurotrauma Neurorepair and Regeneration in Central Nervous System Ministry of Education in China and Tianjin Tianjin Neurological Institute Tianjin Medical University General Hospital Tianjin China; ^5^ Department of Cardiology Tianjin Medical University General Hospital Tianjin China

**Keywords:** D‐dimer, infective endocarditis, ischemic stroke

## Abstract

**Background:**

Ischemic stroke is one of the most prominent and serious neurological complications of infective endocarditis (IE). Our study was designed to evaluate the predictive value of higher level of plasma D‐dimer on admission for the development of ischemic stroke in patients with IE.

**Methods:**

In this prospective study, a total of 173 consecutive patients with IE were recruited from January 2016 to December 2018. Plasma D‐dimer and other clinical indexes of IE patients were measured after admission. The number of patients who developed ischemic stroke during 6‐month follow‐up was recorded, as well as the occurrence time of ischemic stroke.

**Results:**

Ischemic stroke was observed in 38 (22%) patients during 6‐month follow‐up since definite diagnosis of IE. Patients with ischemic stroke had significantly higher levels of plasma D‐dimer than those of patients without stroke (4982 vs 2205 μg/L, *P* < .001). In addition, *Staphylococcus aureus* infection (HR: 1.96, 95% CI: 1.51‐2.42), mitral valve vegetation (HR: 1.52, 95% CI: 1.32‐1.75), and higher levels of on‐admission plasma D‐dimer (HR: 1.35, 95% CI: 1.27‐1.43) were significantly associated with ischemic stroke. Moreover, D‐dimer levels ≥3393 μg/L served as a strong predictor for ischemic stroke in patients with IE, and the sensitivity and specificity were 78% and 83%, respectively.

**Conclusion:**

Our study suggested that higher level of D‐dimer on admission was an independent predictor for ischemic stroke in patients with IE. These patients may require special attention, in particular within the first trimester after IE diagnosis.

## 1 INTRODUCTION

Infective endocarditis (IE) is a life‐threatening disease, especially in developing countries such as China. The prevalence of IE ranges from 1.5 to 11.6 cases per 100 000 people around the world,^1^ and IE is associated with economic and medical conditions. IE may present many extra‐cardiac symptoms or signs, in which neurological complications are the most prominent and severe.^2^ The manifestations of neurological complications include ischemic stroke, hemorrhagic stroke, mycotic aneurysms, brain abscesses, meningitis, and encephalitis.^3^

Ischemic stroke, characterized by the interruption of cerebral blood flow, is a dominant and frequent neurological complication of left‐sided IE 4 as well as the main stroke type among all stroke cases in IE. On the basis of a retrospective study involving eight centers in Spain, 192 (14%) of 1345 consecutive left‐sided IE patients experienced ischemic stroke.^5^ However, some clinicians claimed that the actual incidence of ischemic stroke in IE was much higher than that previously reported because of the existence of asymptomatic stroke. Ischemic stroke has a major influence on the quality of life of IE patients and causes a significant increase in medical expenses. Moreover, ischemic stroke is one of the most important risk factors that will impact the prognosis and increase the mortality of IE.^6^ Though many studies have focused on the risk factors and prevention of ischemic stroke at the early stage of IE, the identification of high‐risk patients remains a challenge.

Plasma D‐dimer, as a degradation product of cross‐linked fibrin, is generated during the activation process of coagulation and the fibrinolysis system.^7^ Plasma D‐dimer is generally seen as a traditional biomarker of thrombotic or embolic diseases, such as deep vein thrombosis or pulmonary embolism. In a cohort study, Folsom et al^8^ reported that a higher level of D‐dimer was a risk factor for ischemic stroke, especially cardioembolic stroke. Further, many studies have reported that elevated levels of D‐dimer were associated with poorer prognosis and functional outcome in patients with ischemic stroke.^9^, ^10^

In a retrospective study, Bakal et al^11^ observed increased levels of D‐dimer in IE patients who experienced significant systemic embolism. However, no prior research has yet evaluated D‐dimer as a potential risk factor for ischemic stroke in IE patients who were treated with conservative medical therapy. The purpose of our study was to investigate whether increased levels of plasma D‐dimer could predict the development of ischemic stroke in nonsurgical IE patients.

## 2 SUBJECTS AND METHODS

### 2.1 Study population

In this prospective study, a total of 189 consecutive IE patients were enrolled from January 2016 to December 2018 derived from Tianjin General Hospital. All patients were hospitalized for the first time with a definite diagnosis of IE based on the modified Duke criteria.^12^ General clinical profiles of IE patients, such as age, gender, predisposing factors, and comorbid diseases, were recorded for further analyses.

Patients who underwent cardiac surgery during admission or follow‐up were excluded considering the increased risk of stroke during and subsequent to surgery.^13^ Patients with a history of any stroke or transient ischemic attacks any time prior to admission were excluded. Those with pacemakers that were not suitable for undergoing brain MRI were excluded. Those on anticoagulant therapy or with coagulation disorders were excluded. Patients with right‐sided IE were also excluded from the study.

The ethics committee of our hospital confirmed the study protocol. Signed informed consent forms were collected from all participators. Our study complied with the principles elaborated in the Declaration of Helsinki.

### 2.2 Echocardiography

All patients underwent standard transthoracic echocardiography (TTE) assessments conducted by a professional cardiologist after admission. Transesophageal echocardiography was carried out in the following situations: unsatisfactory image quality of TTE, clinically suspected prosthetic valve endocarditis, and strong suspicion of IE despite negative TTE findings. Vegetation size was measured in various dimensions, and the maximum diameter was recorded when the oscillating mass appeared the largest.

### 2.3 Diagnosis of ischemic stroke

The primary diagnosis of ischemic stroke was mainly based on clinical neurological symptoms and imaging manifestations on brain MRI. Brain MRI was performed by applying a 1.5‐Tesla GE system with a standardized imaging protocol. An acute ischemic lesion was identified as a hyperintense signal with restricted apparent diffusion on diffusion‐weighted imaging. Ischemic stroke associated with IE may present a variety of patterns, from single lesion to multiple small or large lesions in multiple vascular territories. A definite diagnosis of ischemic stroke was confirmed by an experienced neurologist who was blinded to the clinical data, laboratory examinations, and MRI images.

### 2.4 Laboratory examinations

Measurement of plasma D‐dimer on admission was performed using the VIDAS D‐dimer assay (bioMérieux), which has shown comparable sensitivity and specificity.^14^ The normal range of plasma D‐dimer is 0‐500 μg/L. Other hematological and biochemical data, such as white blood cell (WBC) count and C‐reactive protein (CRP), were also detected from peripheral blood samples collected on the next morning after admission. In addition, to identify causative organisms, at least three sets of blood cultures were obtained from all patients before the initiation of antibiotic therapy.

### 2.5 Follow‐up

Merkler et al^15^ demonstrated that short‐term risk of ischemic stroke increased in the fourth month before IE diagnosis, peaked in the first month after IE diagnosis, then declined rapidly, and stabilized at a general level by the sixth month after diagnosis. Therefore, we selected 6 months as the duration of follow‐up to analyze the temporal relationship between ischemic stroke and IE.

In our study, all patients were followed up from the establishment of IE diagnosis until the occurrence of ischemic stroke or 6‐month follow‐up. The clinical data were recorded for further analyses.

### 2.6 Statistical analysis

All statistical analyses were performed with SPSS (Version 22.0, Chicago, USA). Continuous variables are shown as the mean ± standard deviation or median (interquartile). Categorical variables are shown as percentages. The Mann‐Whitney *U* test or *t* test was applied to compare the different outcomes between subgroups. The chi‐squared test was used for comparison of categorical variables. Cox regression was used to analyze the related risk factors for outcome with hazard ratios and the 95% confidence interval (CI). An exploratory evaluation for additional cut points of different variables was performed using receiver operating characteristic (ROC) curve analysis. The hazard curve to assess the D‐dimer level during the 6‐month follow‐up was analyzed using the Kaplan‐Meier method, and the difference between curves was compared using the log‐rank test. A *P* value < .05 was considered to be statistically significant.

## 3 RESULTS

### 3.1 Baseline characteristics of patients

A total of 189 consecutive IE patients were enrolled in the study, and 173 patients (60% males, mean age, 55.1 ± 13.8 years) completed the follow‐up. Thirty‐eight (22%) patients developed ischemic stroke during the 6‐month follow‐up period and were defined as the stroke group. The remaining 135 (78%) patients without ischemic stroke were classified as the non‐stroke group. A total of 158 (91%) patients suffered from native valve endocarditis, while 15 (9%) patients had prosthetic valve endocarditis. Vegetations were observed in 169 (98%) patients based on the evidence from transthoracic or transesophageal echocardiography. Single‐position vegetations were identified in 149 (86%) patients, most often found at the mitral valve, followed by the aortic valve. Positive results of blood cultures were obtained from 107 (62%) patients. *Streptococcus* identified in 40 (23%) patients was the most common microorganism, accounting for 37% of all positive blood cultures. The secondary dominant causative agent was *Staphylococcus aureus*, isolated in 33 (19%) patients and 31% of all positive blood cultures. The clinical and demographic characteristics of the study population are summarized in Table 1.

**Table 1:** Baseline characteristics of patients with IE

Variables	Total (n = 173) (100%)	Stroke (n = 38) (22%)	Non‐stroke (n = 135) (78%)	*P* value
Age (y)	55.1 ± 13.8	53.9 ± 13.2	56.8 ± 15.3	.78
Male, n (%)	103 (60%)	24 (63%)	79 (59%)	.59
Smoking, n (%)	96 (55%)	22 (58%)	74 (55%)	.76
Coronary heart disease, n (%)	21 (12%)	4 (11%)	17 (13%)	.89
Degenerative valve disease, n (%)	22 (13%)	6 (16%)	16 (12%)	.63
Congenital heart disease, n (%)	9 (5%)	2 (5%)	7 (5%)	.98
Rheumatic heart disease, n (%)	28 (16%)	8 (21%)	20 (15%)	.55
Hypertension, n (%)	27 (16%)	5 (13%)	22 (16%)	.81
Diabetes, n (%)	38 (22%)	7 (18%)	31 (23%)	.68
Atrial fibrillation, n (%)	16 (9%)	5 (13%)	11 (8%)	.46
Native valve involvement				
Mitral valve, n (%)	80 (46%)	22 (58%)	58 (43%)	<.001
Aortic valve, n (%)	87 (50%)	17 (45%)	70 (52%)	.18
Prosthetic valve involvement	15 (9%)	5 (13%)	10 (7%)	.57
White blood cell count (×10^9^/L)	10.9 ± 3.2	11.6 ± 3.4	10.7 ± 2.9	.33
Total cholesterol (mmol/L)	4.3 ± 1.6	4.5 ± 1.8	4.2 ± 1.3	.81
Total triglycerides (mmol/L)	1.6 ± 0.5	2.1 ± 0.6	1.3 ± 0.5	.39
HDL (mmol/L)	1.4 ± 0.6	1.2 ± 0.8	1.5 ± 0.5	.55
LDL (mmol/L)	2.4 ± 1.0	2.6 ± 1.2	2.3 ± 1.0	.80
D‐dimer (μg/L)	2815 ± 1886	4982 ± 2159	2205 ± 1253	<.001
CRP (mg/L)	59 ± 15	67 ± 18	52 ± 13	.02
Blood cultures				
*Staphylococcus aureus*	33 (19%)	11 (29%)	22 (16%)	<.001
Coagulase‐negative *staphylococcus*	13 (8%)	3 (8%)	10 (7%)	.75
*Streptococcus*	40 (23%)	8 (21%)	32 (24%)	.52
Others^a^	21 (12%)	3 (8%)	18 (13%)	.46
Culture‐negative	66 (38%)	13 (34%)	53 (39%)	.67
Vegetation size > 10 mm	82 (47%)	20 (53%)	62 (46%)	.71

Note

Data are expressed as mean ± SD or number (percentage).

Abbreviations: CRP, C‐reactive protein; HDL, high‐density lipoprotein; LDL, low‐density lipoprotein; *S aureus, Staphylococcus aureus*.

a *Enterococcus*, Gram‐negative bacilli.

There was no significant difference between the stroke and non‐stroke groups in terms of age, gender, and comorbid diseases such as hypertension, diabetes, or underlying heart diseases. However, the on‐admission D‐dimer levels of IE patients who developed ischemic stroke were significantly higher than for those without stroke during the follow‐up (4982 vs 2205 μg/L, *P* < .001). Moreover, a comparison of CRP levels showed a similar result for D‐dimer levels between the two groups (67 vs 52 mg/L, *P* = .02).

The incidence of mitral valve vegetation in the stroke group was significantly higher than that in the non‐stroke group (58% vs 43%, *P* < .001). In addition, patients with ischemic stroke had a higher prevalence of *S aureus* infection (29% vs 16%, *P* < .001). Moreover, patients with ischemic stroke more often had vegetation sizes >10 mm, though there was no statistically significant difference (53% vs 46%, *P* = .71).

**Figure 1:**
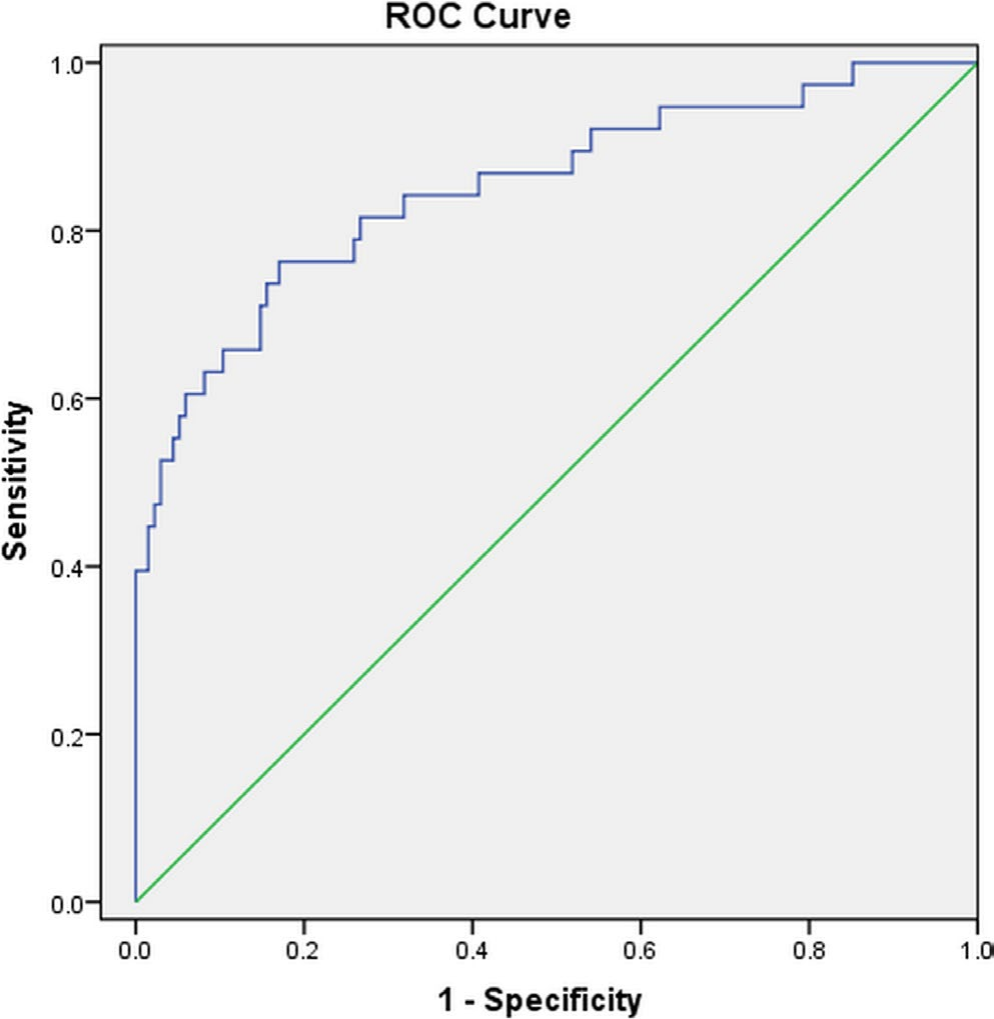
Receiver operating characteristic (ROC) curve of D‐dimer level for predicting ischemic stroke. The ROC analysis displays the specificity and sensitivity of D‐dimer level for ischemic stroke prediction

Receiver operating characteristic (ROC) curves were performed to explore the relation between plasma D‐dimer levels on admission and ischemic stroke during the 6‐month follow‐up. The area under the curve (AUC) was 0.856 (95% CI: 0.77‐0.92; *P* < .001). On admission, plasma D‐dimer levels over a cutoff level of 3393 μg/L predicted ischemic stroke well during the 6‐month follow‐up, with a sensitivity of 78% and a specificity of 83% (Figure 1).

**Figure 2:**
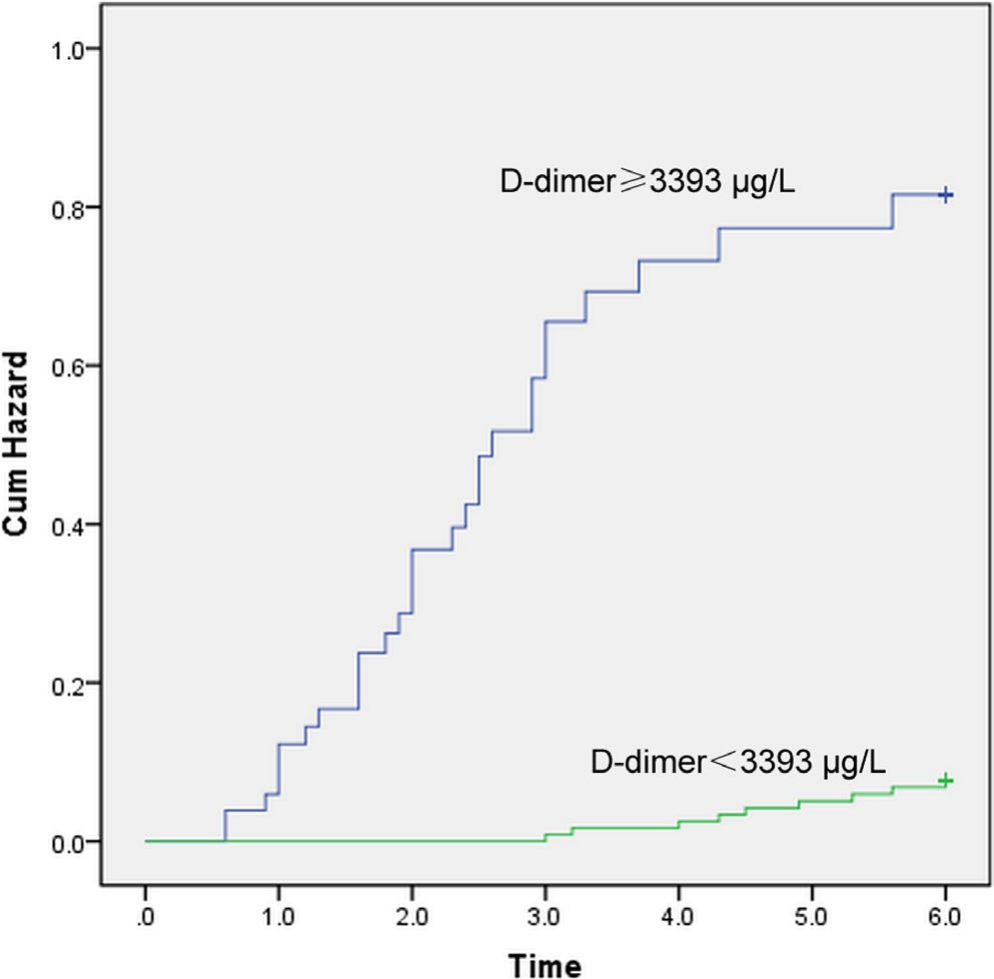
Kaplan‐Meier curve for the cumulative incidence of ischemic stroke in patients with IE. The patients were stratified by D‐dimer cutoff level of 3393 μg/L

According to the D‐dimer cutoff level of 3393 μg/L, all IE patients were classified into two subgroups, and the Kaplan‐Meier curve was plotted for the cumulative incidence of ischemic stroke during the 6‐month follow‐up (Figure 2). The results showed that D‐dimer levels ≥3393 μg/L were significantly associated with the incidence of ischemic stroke (*P* < .001).

Cox regression was applied to analyze the predictors of ischemic stroke in IE patients. In univariate analysis, *S aureus* infection, mitral valve vegetation, higher CRP levels, higher D‐dimer levels, D‐dimer levels ≥3393 μg/L, and vegetation sizes >10 mm were significantly associated with ischemic stroke. When multivariate Cox regression was applied after adjusting for other confounders, *S aureus* infection (HR: 1.96, 95% CI: 1.51‐2.42), mitral valve vegetations (HR: 1.52, 95% CI: 1.32‐1.75), higher D‐dimer levels (HR: 1.35, 95% CI: 1.27‐1.43), and D‐dimer levels ≥3393 μg/L (HR: 2.03, 95% CI: 1.21‐2.89) were identified as independent predictors of ischemic stroke in IE patients (Table 2).

**Table 2:** Univariate and multivariate Cox regression analyses for ischemic stroke

Variables	Univariate analysis		Multivariate analysis	
	HR (95% CI)	*P* value	HR (95% CI)	*P* value
Age	1.02 (0.95‐1.12)	.659		
Gender (Male)	1.05 (0.76‐1.62)	.701		
*Staphylococcus aureus* infection	2.02 (1.37‐2.53)	<.001	1.96 (1.51‐2.42)	<.001
WBC count	1.03 (1.02‐1.09)	.106		
CRP	1.09 (1.06‐1.18)	.022	1.02 (0.98‐1.13)	.237
D‐dimer	1.39 (1.13‐1.68)	<.001	1.35 (1.27‐1.43)	<.001
D‐dimer > 3393	2.56 (1.38‐3.76)	<.001	2.03 (1.21‐2.89)	<.001
Vegetation size > 10 mm	1.16 (1.08‐1.26)	.026	1.07 (1.02‐1.13)	.102
Mitral vegetation	1.56 (1.37‐1.78)	<.001	1.52 (1.32‐1.75)	<.001

Note

Abbreviations: CI, confidence interval; HR, hazard ratio.

## 4 DISCUSSION

Early and accurate identification of high‐risk IE patients who may develop ischemic stroke will give clinicians the chance for prompt intervention and more active therapy. Several studies have assessed possible risk factors for IE on admission and correlative risk of ischemic stroke.

García‐Cabrera et al^5^ claimed that mitral valve involvement in IE was a risk factor that could not be ignored for ischemic stroke. According to a retrospective study by Cao et al,^16^ the odds ratio was 1.648 for stroke in IE patients with mitral valve vegetation. In accordance with the aforementioned results, our study also identified mitral valve vegetation as an independent risk factor for ischemic stroke in IE patients. Therefore, it is reasonable to consider that mitral valve vegetation plays an important role in the development of ischemic stroke.

Based on a retrospective study by Valenzuela et al,^17^ *Staphylococcus* infection was one of the most important risk factors for ischemic stroke. According to the study by García‐Cabrera et al,^5^ *S aureus* was clearly related to 27% of ischemic complications as a main causative organism. Considering the fact that many patients already received oral or venous antibiotic treatment prior to admission, which lowered the positive rate of blood cultures, the actual prevalence of *S aureus* infection may be underestimated in our study. However, *S aureus* was still a dominant pathogen, accounting for 44% of positive blood cultures in the stroke group.

Our findings were consistent with previous studies regarding the predictive roles of mitral valve vegetation and *S aureus* infection. However, there was no statistically significant difference between the stroke and non‐stroke groups in terms of vegetation size >10 mm. According to the 2015 ESC guidelines for the management of IE, increasing vegetation size was considered to be related to an increased risk of embolism.^18^ Vilacosta et al^19^ reported that patients with vegetation sizes >10 mm had a higher incidence of embolic events when staphylococcal endocarditis and the mitral valve were involved. In our study, patients with larger vegetation sizes were more likely to undergo cardiac surgery during hospitalization or follow‐up due to the development of heart failure or uncontrolled infection or the prevention of embolic events. In fact, after the exclusion of these patients from the study, the actual incidence of ischemic stroke in patients with vegetation sizes >10 mm would be underestimated, contributing to the similar consequences in stroke and non‐stroke groups.

As discussed above, mitral valve vegetation and *S aureus* infection were independent predictors of ischemic stroke in IE patients. However, it is worth exploring more simple and convenient risk factors that could help us to identify high‐risk patients at an early stage.

This study aimed to assess the relation between on‐admission D‐dimer levels and the risk of ischemic stroke in IE patients. To the best of our knowledge, few studies have explored the association between higher levels of D‐dimer and the development of ischemic stroke in IE patients. With up to 6‐month follow‐up, our study revealed that higher levels of D‐dimer were associated with an increased risk of ischemic stroke, and D‐dimer levels ≥3393 μg/L could be seen as a strong predictor for ischemic stroke. This association remained significant after adjusting for other interference factors.

Further, the temporal relationship between the diagnosis of IE and the occurrence of ischemic stroke was also illuminated in our study. According to the D‐dimer cutoff level of 3393 μg/L, the occurrence time of ischemic stroke was categorized and shown in Table 3. Twenty‐five (86%) patients developed ischemic stroke during the first trimester after a definite diagnosis of IE when the D‐dimer level on admission was ≥3393 μg/L.

**Table 3:** Occurrence time of ischemic stroke categorized by D‐dimer cutoff level

Months	D‐dimer ≥ 3393 μg/L (n = 29)	D‐dimer < 3393 μg/L (n = 9)
0‐1	6 (21%)	0 (0%)
1‐2	10 (34%)	0 (0%)
2‐3	9 (31%)	1 (11%)
3‐4	2 (7%)	2 (22%)
4‐5	1 (3%)	3 (33%)
5‐6	1 (3%)	3 (33%)
Total	29	9

Our study may provide future reference for clinicians in some aspects. On the one hand, higher levels of D‐dimer on admission could help us screen high‐risk IE patients for ischemic stroke. On the other hand, our study may offer insights into future positive strategies in the prevention of ischemic stroke in IE.

Cerebral embolism from cardiogenic vegetation is generally recognized as a main mechanism responsible for ischemic lesions in IE.^4^ However, the underlying association between higher levels of D‐dimer and the risk of cardioembolic stroke is still unknown. Previous study proved that hyperactivity of the coagulation and fibrinolysis system was highlighted during the acute stage of cardioembolic stroke.^20^ In IE patients, vegetation resulted from the infection of pathogenic microorganism and aggregation of platelet. Afterward, cytokines such as IL‐1, IL‐6, and TNF‐α were released, and then, coagulation cascade was activated,^21^ resulting in an elevated level of D‐dimer.^22^ So, a possible mechanism may be that elevated D‐dimer level is a reflection of currently unclear thrombotic and hemostatic disorders associated with the development of cardioembolic stroke.^23^ In addition, D‐dimer is also a biomarker of inflammatory processes.^24^ Research has shown that inflammation contributed to the development of ischemic lesions in patients with IE.^25^

Therefore, instead of the cause, elevated D‐dimer level might be a marker of mechanisms related to the development of ischemic stroke.^23^ More studies are needed to illuminate the specific pathophysiological processes underlying the complicated relationship between D‐dimer and ischemic stroke in IE.

Our study had some limitations. First, our study was a single‐center study with a relatively smaller sample size. Therefore, the results need to be verified further in a larger‐scale study. Second, the actual incidence of ischemic stroke may be underestimated in our study. Okazaki et al^25^ evaluated 85 patients with left‐sided IE who underwent cardiac surgery. Preoperative brain MRI discovered that acute ischemic lesions existed in 47 (55%) patients. However, only 19 (22%) patients showed typical neurological symptoms, suggesting that a majority of patients developed asymptomatic strokes. Considering the fact that neurological symptoms were important clues and the basis for the diagnosis of ischemic stroke, a certain number of asymptomatic patients who developed ischemic stroke may be ignored in our study. However, it is still controversial that all IE patients undergo neuroimaging examination regardless of the presence or absence of neurological symptoms.

Our study revealed that higher levels of on‐admission plasma D‐dimer showed high sensitivity and specificity in the prediction of ischemic stroke, which occurred mostly in the first trimester after the diagnosis of IE. On the one hand, as a traditional and inexpensive biomarker, plasma D‐dimer may help clinicians identify high‐risk IE patients for ischemic stroke in a simple and quick way. On the other hand, these patients may need special attention as well as appropriate prevention and treatment strategies. Therefore, a higher level of D‐dimer may be a valuable predictor for ischemic stroke in IE patients, as well as an important reference factor in risk stratification and preventive strategy establishment.

## CONFLICT OF INTEREST

No potential conflict of interest was reported by the authors.
